# Impact of preanalytical factors on blood CHI3L1 levels

**DOI:** 10.3389/fimmu.2025.1720914

**Published:** 2026-02-02

**Authors:** Manuel Comabella, Lucía Gutierrez, Mireia Castillo, Luisa M. Villar, Herena Eixarch, Delon La Puma, Montserrat Aroca, Andreu Vilaseca, Xavier Montalban, Nicolás Fissolo

**Affiliations:** 1 Servei de Neurologia and Centre d’Esclerosi Múltiple de Catalunya (Cemcat), Institut de Recerca Vall d’Hebron (VHIR), Hospital Universitari Vall d’Hebron, Universitat Autònoma de Barcelona, Barcelona, Spain; 2 Center for Networked Biomedical Research on Neurodegenerative Diseases (CIBERNED) - ISCIII, Madrid, Spain; 3 Departments of Neurology and Immunology , Hospital Universitario Ramón y Cajal, Instituto Ramón y Cajal de Investigación Sanitaria, Madrid, Spain; 4 Universitat de Vic-Universitat Central de Catalunya (Uvic-UCC), Vic, Catalonia, Spain

**Keywords:** biomarkers, chitinase 3-like 1, multiple sclerosis, preanalytical factors, single molecule array

## Abstract

**Background:**

Chitinase 3-like 1 (CHI3L1) is a prognostic biomarker in multiple sclerosis (MS). However, its clinical application is limited by a lack of standardized detection methods and concerns about preanalytical variability.

**Objectives:**

This study aims to evaluate the impact of preanalytical factors (delayed processing of blood and repeated thawing/freezing) on serum CHI3L1 levels. Additionally, we sought to correlate CHI3L1 blood and cerebrospinal fluid (CSF) levels and identified its cellular source in peripheral blood mononuclear cells (PBMCs) from MS patients.

**Methods:**

We used an in-house Single Molecule Array (Simoa) assay to measure CHI3L1 levels in serum, plasma, and CSF from MS patients and controls. The source of CHI3L1 production in PBMCs was determined by flow cytometry.

**Results:**

A strong correlation was found between serum, plasma, and CSF CHI3L1 levels. Serum CHI3L1 levels remained stable with delayed processing up to 6 hours and for up to three freeze-thaw cycles. Monocytes, particularly classical monocytes (CD14^++^CD16^-^ cells), were identified as the main producers of CHI3L1 in PBMCs.

**Conclusions:**

The study establishes preanalytical guidelines for sCHI3L1 assessment and confirms that blood levels can be as informative as CSF levels. This provides groundwork for the standardized use of CHI3L1 as a biomarker in managing MS patients.

## 1 Introduction

Chitinase 3-like 1 (CHI3L1) is a well-established prognostic biomarker in early phases of multiple sclerosis (MS). Several studies performed in cerebrospinal fluid (CSF) samples have shown that elevated CHI3L1 levels are associated with conversion from clinically isolated syndrome (CIS) to MS, as well as with an increased risk of developing disability in these patients (1–3). Moreover, CHI3L1 has been demonstrated to serve as an independent predictor of conversion to secondary progressive MS (SPMS) (4), and also has the potential to predict disability progression in primary progressive MS (PPMS) patients (5).

Unlike other biomarkers whose expression is more CNS specific, CHI3L1 is also produced by a broad range of cells from peripheral compartments, from which CHI3L1 is released to blood (6). This had led to concerns about using serum CHI3L1 as a direct replacement for CSF. Although there are no studies evaluating the correlation between CHI3L1 levels in CSF and blood in MS, our group has demonstrated that CHI3L1 levels in serum and CSF do correlate in people with radiologically isolated syndrome (7). In this regard, several studies have shown that blood levels have also proven useful in assessing disease progression and activity in MS, showing significantly higher concentrations in SPMS and PPMS patients compared to RRMS and healthy controls (8), and predicting disability progression in patients with PPMS (9).

Understanding the preanalytical variability of a biomarker is essential for its implementation in clinical practice. It is known that up to 70% of laboratory errors arise from preanalytical variation (10). Variability in factors related with sample collection, processing, handling and storage contributes to inconsistent biomarker results (11). Therefore, before clinical use it is necessary to assess how susceptible a biomarker is to preanalytical variation in order to establish standard operating procedures that minimize variation and bias from relevant preanalytical factors.

The aim of our study is to determine the impact of the main preanalytical factors on serum CHI3L1 levels. Specifically, we tested the effect of the timing of sample processing, and repeated thawing/freezing. In addition, we assessed the correlation between the levels of CHI3L1 in serum, plasma and CSF, using an *in-house* assay developed on the Single Molecule Array (SIMOA) platform. In a second part of the study, we investigated the cell source of CHI3L1 in peripheral blood samples from patients with MS.

## 2 Methods

### 2.1 Participants and sample handling

Three different cohorts of patients and healthy controls (HC) were included in the study:

(i) Sample correlation analysis were performed in samples from 30 MS patients, based on the availability of paired serum, plasma and CSF samples never thawed.

(ii) Delayed processing time experiments were performed in serum from 17 HC whose blood sample processing (i.e., centrifuging and freezing) was delayed up to 2, 4, 6, and 24 h.

(iii) Freeze-thaw experiments were performed with 28 MS serum samples. To ensure consistency, each freeze-thaw cycle after the first was standardized to an 8-h thaw at room temperature followed by a 16-h freeze at −80 °C.

None of the patients and controls included in the study received treatment with corticosteroids or immunomodulatory and/or immunosuppressive therapies before sample collection. The MS cohorts included both relapsing and progressive disease courses. The study was approved by the Clinical Research Ethics Committee at Vall d’Hebron University Hospital. All participants gave written informed consent.

### 2.2 CSF, serum and plasma processing

CSF samples were collected by lumbar puncture for routine CSF diagnostics, centrifuged to remove cells, and aliquoted. Peripheral blood was collected by standard venipuncture. Serum was obtained by centrifugation after spontaneously clotting for 30 minutes. Plasma samples were equally processed without waiting for the material to coagulate before centrifugation. All samples were stored frozen at −80 °C until used.

### 2.3 Biomarker determinations

To measure CHI3L1 levels, a Simoa-based assay was developed based on the use of a Simoa homebrew assay starter kit as previously described (7). Samples were run in duplicate on the fully automated ultrasensitive Simoa HD-X Analyzer (Quanterix, Billerica, Massachusetts, USA). The intra-assay and inter-assay coefficients of variation were <8% and 12%, respectively. All samples for each preanalytical condition studied were analyzed together using a single reagent batch.

### 2.4 CHI3L1 expression by flow cytometry

CHI3L1 expression was determined by flow cytometry in peripheral blood mononuclear cells (PBMCs) from ten untreated MS patients. The following monoclonal antibodies were used for cell surface staining: anti-CD3-FITC, anti-CD14-APC, anti-CD19-APC-H7, anti-CD16-PerCP-Cy5.5, anti-CD56-BV510, anti-CD45-BV605, and the corresponding isotype controls. For intracellular staining, a polyclonal rabbit antibody anti-human CHI3L1 (#4815, Quidel, San Diego, California, USA) and a purified rabbit IgG (Abcam, Cambridge, MA) were biotinylated with sulfo-NHS-biotin (Thermo Fisher Scientific, San Jose, CA, USA) following the manufacturers’ instructions. Previous intracellular labelling, cells were washed in saline and resuspended in 200 ml of Cytofix/CytopermTM (BD Biosciences, San Diego, CA). After 20 min at 4°C, cells were washed in Perm/Wash buffer, pellets resuspended and labelled with 0.05 mg of anti CHI3L1-biotin or rabbit IgG-biotin for 1 h at 4°C and washed. Finally, cells were incubated with streptavidin-PE (BD Biosciences) for 45 min at 4°C. The specificity of anti-CHI3L1 antibody staining was assessed by means of blocking experiments incubating 0.05 mg of anti-CHI3L1 antibody with 2 mg of human recombinant CHI3L1 protein (Quidel) for 30 min at 4°C before cell labelling. Discrimination of dead cells was achieved by Fixable Viability Stain (Thermo Fisher). Samples were acquired with a CytoFLEX flow cytometer (Beckman Coulter, Brea, CA, USA) and data were analyzed with CytExpert 2.3 software (Beckman Coulter). The list of antibodies and clones used to stain samples is shown on **Supplementary Table 1**.

### 2.5 Statistical analyses

Statistical analysis was conducted using SPSS (Version 22) software (SPSS Inc., Chicago, IL, USA) for MS Windows and GraphPad Prism 10.2.0 software (GraphPad Prism Inc., La Jolla, CA, USA). The distribution of serum, plasma, and CSF CHI3L1 levels was tested for normality with a Kolmogorov-Smirnov test. Afterward, paired and unpaired nonparametric tests were applied for comparisons of mean CHI3L1 levels among groups. Correlations were evaluated with the Spearman test. Differences were considered statistically significant when *P* values were below 0.05.

## 3 Results

### 3.1 CHI3L1 levels in blood correlate with CSF

A good correlation between blood and CSF CHI3L1 levels is essential for the practical application of blood-based measurements to monitor MS patients. To investigate this, we included a group consisting of 30 MS patients whose blood and CSF were obtained in parallel. **Supplementary Table 2** lists characteristics of participants for the correlation analysis and their mean CHI3L1 concentrations in serum (sCHI3L1), plasma (pCHI3L1) and CSF (cCHI3L1) at standard conditions (i.e. processed and frozen immediately and thawed once).

**Figure 1:**
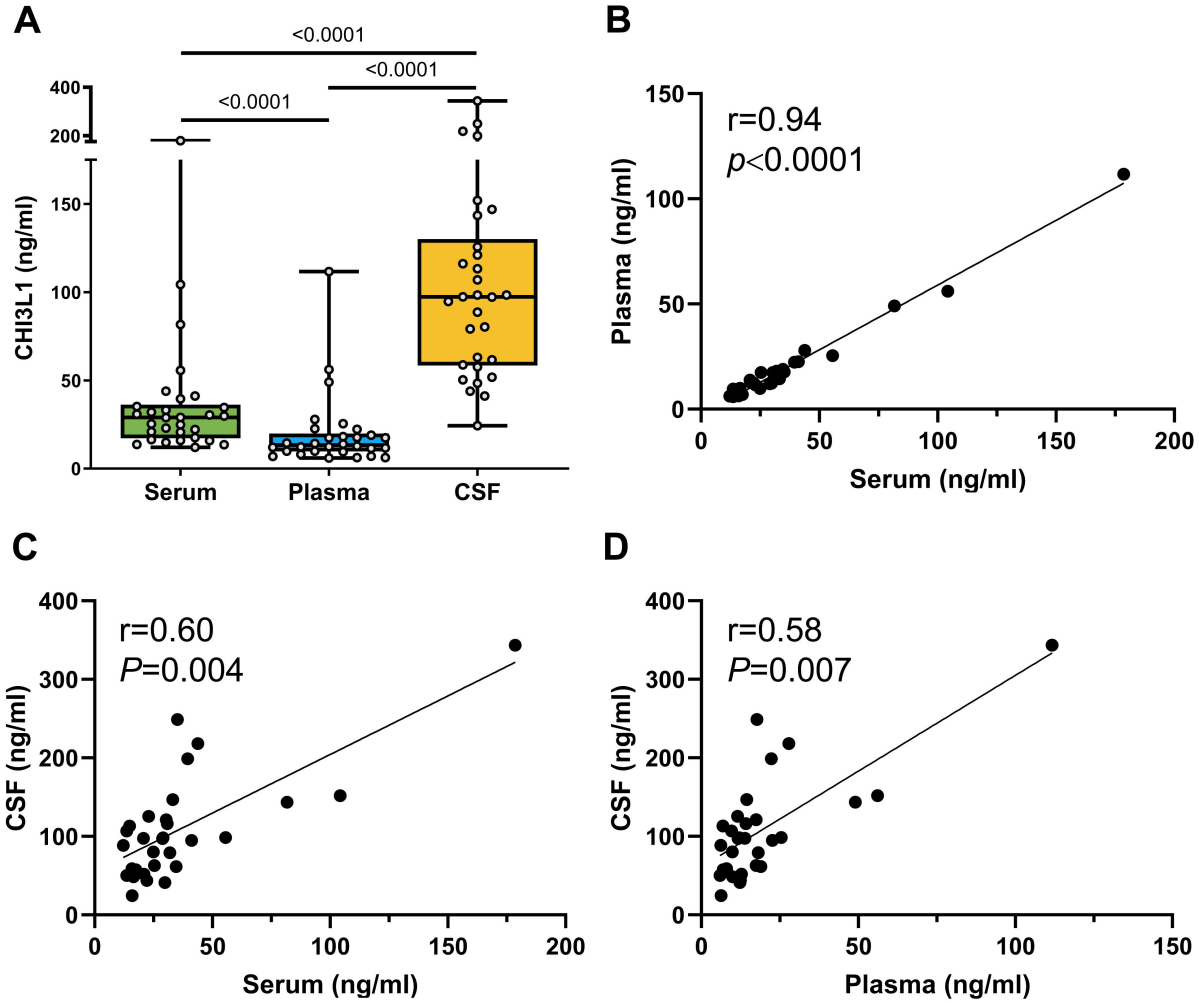
CHI3L1 levels across biological compartments. **(A)** Box plots showing the CHI3L1 levels in paired serum, plasma, and cerebrospinal fluid (CSF) samples. Each dot represents an individual’s measurement (in ng/mL) in all three sample types from the same individuals. A y-axis segmentation was performed to represent better high and low CHI3L1 levels. **(B–D)** Scatter plots showing the Spearman correlation analysis comparing, **(B)** CHI3L1 serum and plasma levels; **(C)** CHI3L1 serum and CSF levels; and **(D)** CHI3L1 plasma and CSF levels. The R values represent Spearman’s rank correlation coefficient. (N = 30).

CHI3L1 levels exhibited a strong correlation between serum and plasma (Spearman r = 0.94; *P* < 0.0001, **Figure 1B**). Significant correlations were also observed between paired CSF and serum (Spearman r = 0.60; *P* < 0.001) (**Figure 1C**), and between CSF and plasma (Spearman r = 0.58; *P* < 0.001, **Figure 1D**). No significant correlations were found between CHI3L1 levels in any of the three compartments and age (Spearman r = 0.10, *P* = 0.6 for serum; Spearman r = 0.20, *P* < 0.3 for plasma; Spearman r = 0.14, *P* = 0.4 for CSF, **Supplementary Figure 1**).

Comparison of CHI3L1 levels across CSF, serum, and plasma demonstrated significantly elevated levels in CSF compared to both blood fractions. Specifically, the overall mean cCHI3L1 concentration was 109.0 ng/mL (95% CI 83.2–134.8), while mean concentrations in blood were 36.18 ng/mL (95% CI 23.7–48.7) for sCHI3L1, and 19.7 ng/mL (95% CI 11.9–27.4) for pCHI3L1. This represents a significant 3-fold increase in CSF levels over serum (*P* < 0.0001) and a 5.5-fold increase over plasma (*P* < 0.0001, **Figure 1A**). Furthermore, within the blood compartment, sCHI3L1 levels were significantly higher than pCHI3L1 levels (P < 0.0001, **Figure 1A**).

### 3.2 CHI3L1 concentrations withstand delayed blood processing up to 6 h

**Figure 2:**
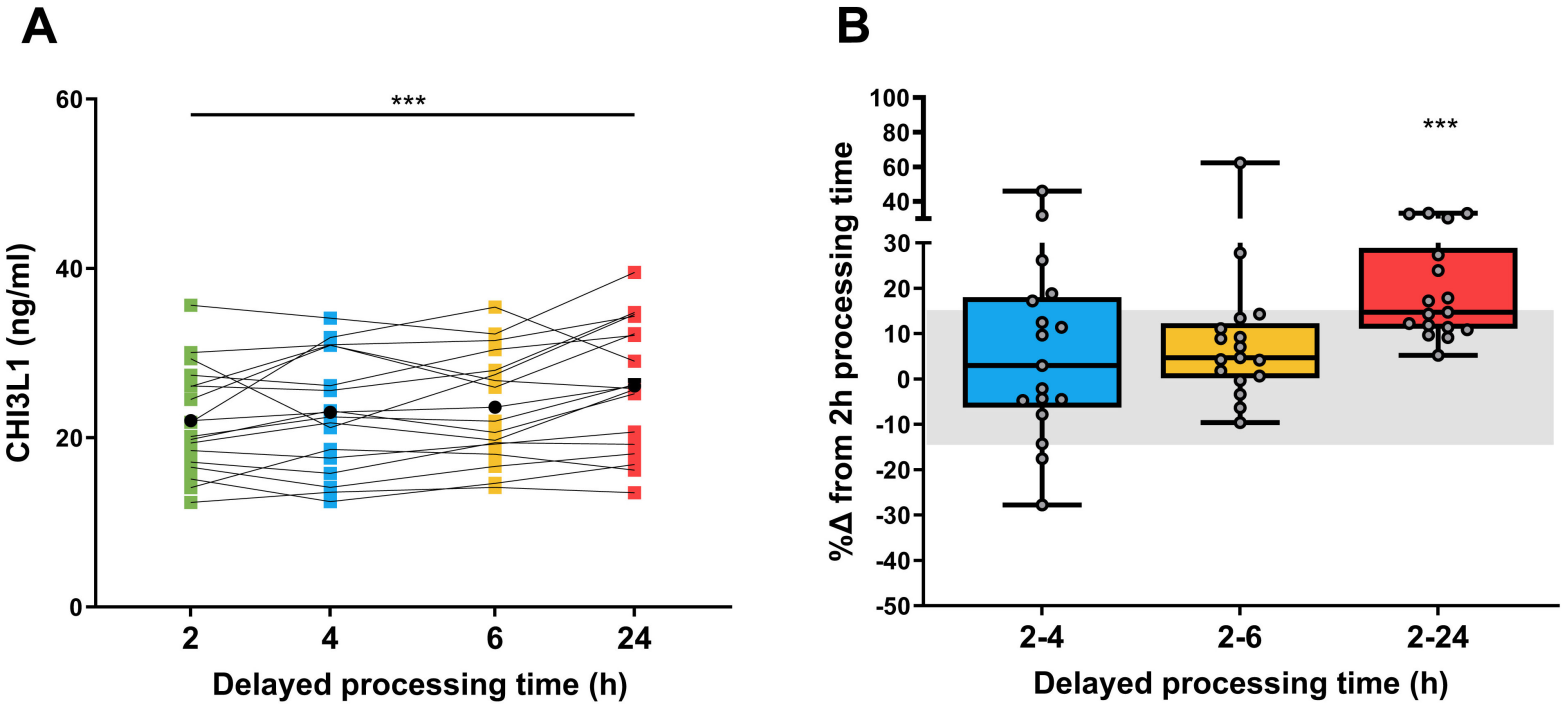
Impact of delayed processing time on sCHI3L1 levels. **(A)** Longitudinal chart depicting the sCHI3L1 concentrations at various processing times: 2, 4, 6, and 24 hours post-blood collection. Individual measurements are shown as colored dots, with black dots indicating the mean value for each processing condition. **(B)** Box plots representing the percentage of change (%Δ) in CHI3L1 levels comparing delayed processing time of 4, 6, and 24 h against the 2h-reference time. Individual values are represented by grey dots, and the shaded grey area represents a change of ±15%. ***P <.001 comparing the 24 h against the 2 h (N = 17).

The impact of time between collection and processing (defined as delayed processing time) was analyzed in blood samples stored at room temperature for 2 (reference), 4, 6, and 24 h before processing. **Figure 2** shows that there was no significant difference in sCHI3L1 levels between samples processed after 2 h, 4 h, or 6 h. The mean concentrations were 22.0 ng/mL (95% CI 18.7–25.3), 23.0 ng/mL (95% CI 19.4–26.6), and 23.6 ng/mL (95% CI 20.3–27.0), respectively (**Figure 2A**, **Supplementary Table 3**). However, when samples were stored for 24 h before processing, a significant increase of 18.8% compared with the reference condition was observed (mean concentration of 26.1 ng/mL vs. 22.0 ng/mL; *P*< 0.0001, **Figure 2A**). **Figure 2B** shows the %Δ in sCHI3L1 levels in samples processed after 4 h, 6 h, and 24 h compared to 2 h (reference).

### 3.3 CHI3L1 concentrations remain stable up to three freeze-thaw cycles

**Figure 3:**
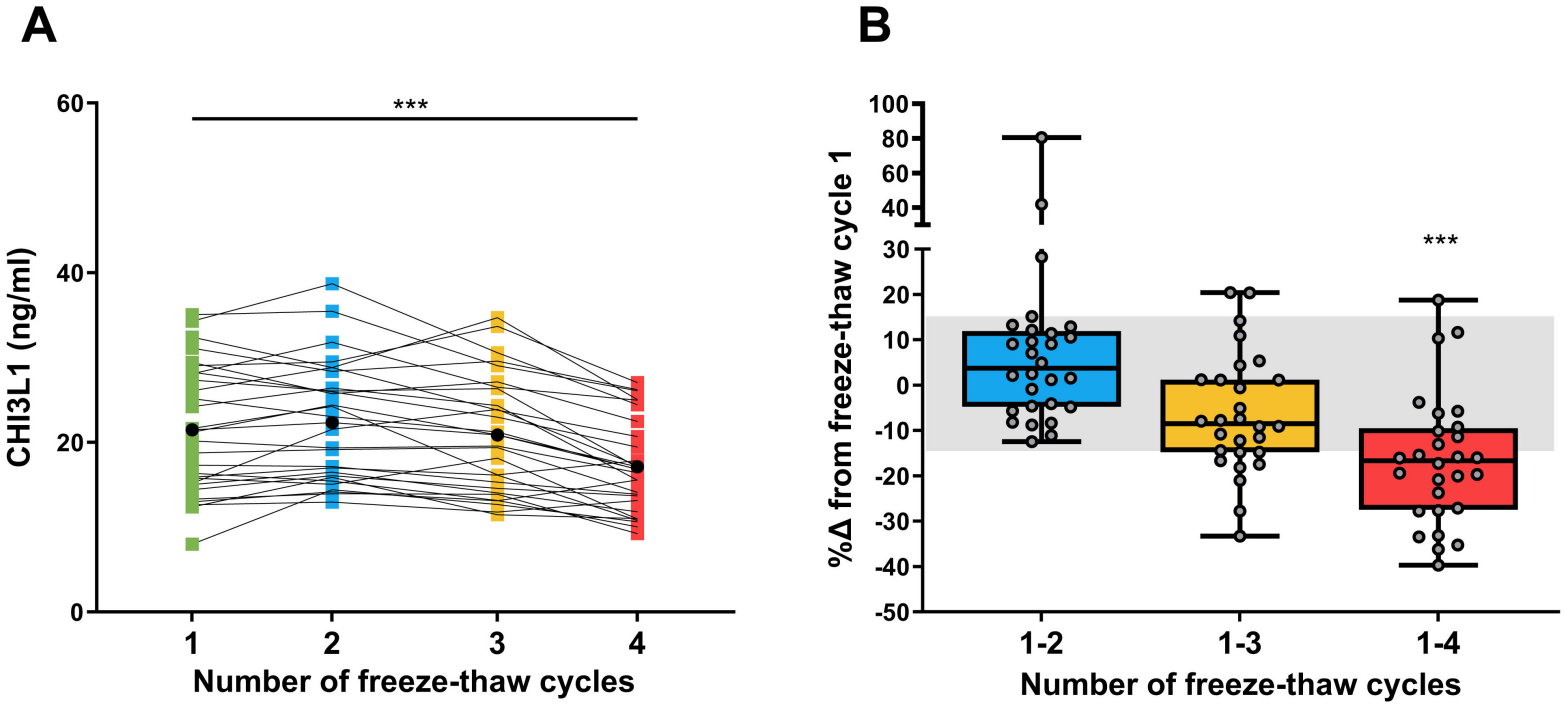
Effect of repeated freeze–thaw cycles on sCHI3L1 levels. **(A)** Longitudinal chart showing the sCHI3L1 levels across four freeze–thaw cycles. Individual measurements are shown as colored dots, with black dots indicating the mean value for each freeze–thaw cycle. **(B)** Box plots representing the percentage of change (%Δ) in CHI3L1 levels. This change is calculated for freeze-thaw cycles 2, 3, and 4, relative to the initial freeze-thaw cycle (cycle 1). Individual values are represented by grey dots, and the shaded grey area represents a change of ±15%. ***P <.001 comparing sCHI3L1 levels in cycle 4 against cycle 1. (N = 28).

We next assessed whether sCHI3L1 levels were sensitive to repeated freeze–thaw cycles. As depicted in **Figure 3**, sCHI3L1 levels were shown to be stable up to freeze-thaw cycle 3. Mean concentrations across the first three cycles were consistent: 21.5 ng/mL (95% CI 18.5–24.4) for cycle 1, 22.3 ng/mL (95% CI 19.6–25.1) for cycle 2, and 20.8 ng/mL (95% CI 18.2–23.5) for cycle 3 (**Figure 3A**, **Supplementary Table 4**). However, a significant decrease in sCHI3L1 concentration was observed at the fourth freeze-thaw cycle (mean: 17.1 ng/mL, 95% CI 14.9–19.3) compared to freeze-thaw cycle 1 *(P*< 0.0001, **Figure 3A**). **Figure 3B** shows the %Δ in sCHI3L1 levels in freeze–thaw cycles 2 to 4 compared to freeze–thaw cycle 1 (reference).

### 3.4 Monocytes are the main producers of CHI3L1 in PBMCs

**Figure 4:**
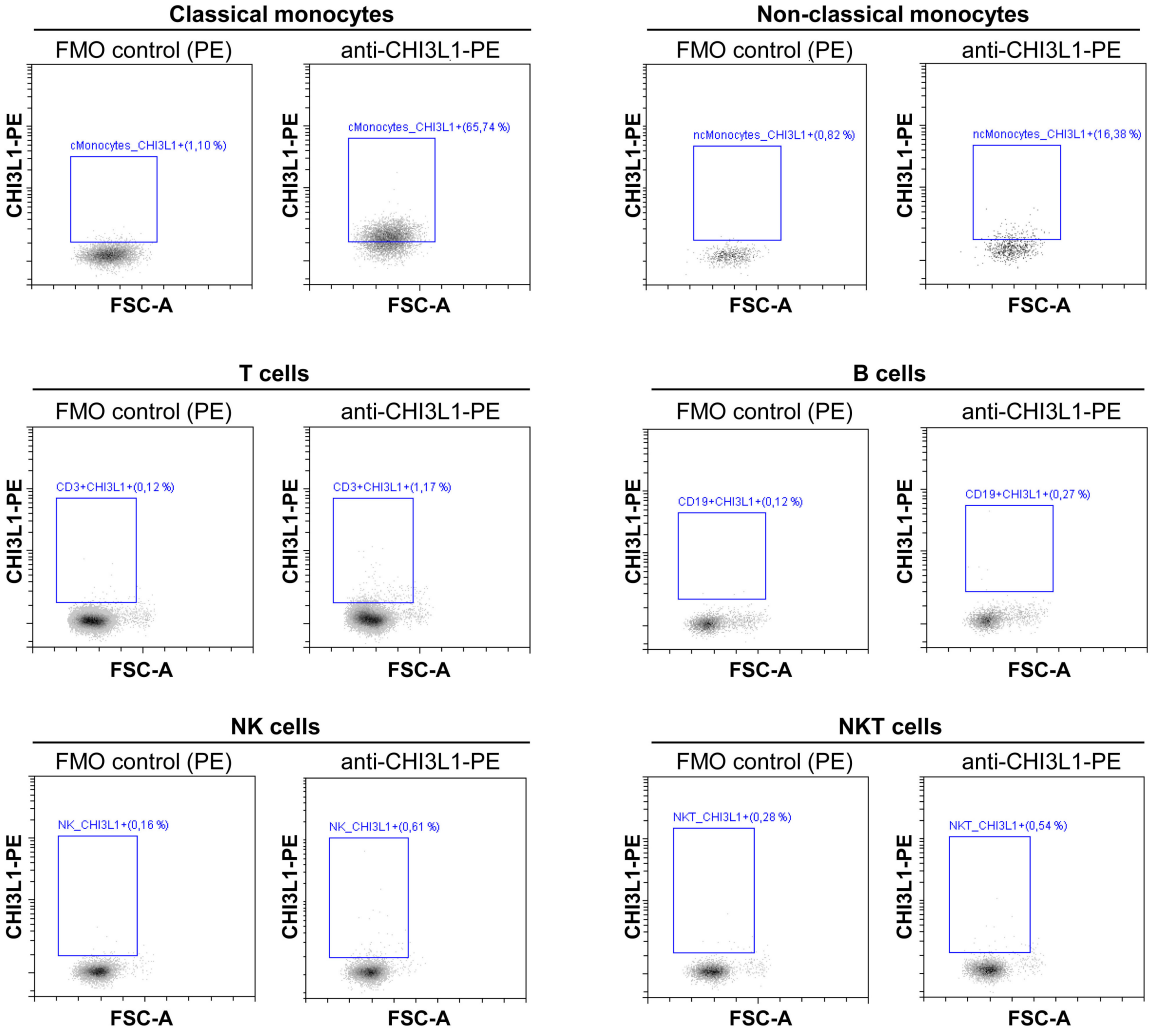
CHI3L1 expression in PBMCs. Intracellular CHI3L1 expression was determined by flow cytometry in peripheral blood mononuclear cells from MS patients. Representative dot plots from one MS patient illustrate CHI3L1 expression across major leukocyte subsets, including: classical monocytes (CD14^++^CD16^-^) non-classical monocytes (CD14^+^ CD16^+^), T cells (CD3^+^), B cells (CD19^+^), NK cells (CD56^+^), and NKT cells (CD56^+^CD3^+^). For each cell population shown, the left panel displays the Fluorescence Minus One (FMO) control for the PE channel, and the right panel presents the anti-CHI3L1-PE stained sample, confirming specific staining. The full gating strategy for subset identification is detailed in **Supplementary Figure 2**. FSC-A: Forward Scatter Area. CHI3L1: chitinase 3 like-1. cMonocytes: classical monocytes. ncMonocytes: non-classical monocytes.

As a last step, we investigated the CHI3L1 cell source by flow cytometry in PBMCs from MS patients. We found that the main cells expressing CHI3L1 were monocytes (CD14^+^). Within this group, classical monocytes (CD14^++^ CD16^-^ cells) showed the highest expression, while non-classical monocytes (CD14^+^ CD16^++^) also expressed the protein, their contribution was less substantial due to their lower numbers within the PBMC population. In contrast, CHI3L1 expression was absent in T (CD3^+^), B (CD19^+^), NK (CD56^+^), and NKT (CD3^+^CD56^+^) cells (**Figure 4**).

## 4 Discussion

To fully assess the clinical potential of CHI3L1 to monitor MS progression, it is critical first to address some key challenges. This includes to translate CSF data to a more accessible sample type as blood; to address the preanalytical factors affecting the serum levels, and to establish a consistent assay method. In the present study we found that blood levels, serum and plasma, strongly correlated with CSF levels, indicating that blood levels could be as informative as CSF. We also observed that a delay in blood processing of 6 h at room temperature, and up to three cycles of freeze-thaw do not have a significant impact on the sCHI3L1 levels. In addition, CHI3L1 levels in CSF and blood can be sensitively and reliably quantified by a novel analytical approach, a homebrew Simoa assay.

In agreement with previous studies, we found that cCHI3L1 levels were significantly higher than those in blood (3). This reinforces the idea that CHI3L1 detected in the CSF had been predominantly produced within the CNS, primarily by astrocytes within active and chronic active lesions of MS brains (12–14), with some contribution from microglia (1), rather than being transported from the bloodstream. In this line, Canto et al. have previously demonstrated the limited ability of CHI3L1 to cross the BBB (1). Interestingly, our flow cytometry experiments identified CD14^+^ monocytes as the primary source of CHI3L1 production in blood compartments, though we cannot exclude the possibility that these monocytes, upon infiltrating the CNS, might also contribute to cCHI3L1 levels, consistent with our group’s previous findings on CHI3L1 expression by monocytes in CSF cells (1). Regarding differences between serum and plasma, previous studies have also shown higher levels of CHI3L1 in serum (15, 16), probably caused by release of CHI3L1 from activated neutrophils during the coagulation process.

Our investigation into preanalytical factors revealed that sCHI3L1 concentrations remained consistent for up to 6 h when blood samples were stored at room temperature, exhibiting levels similar to a 2 h-reference processing time. Conversely, a significant increase in sCHI3L1 was detected following 24 h of delayed processing at room temperature. Similarly, previous studies have also shown increases in CHI3L1 up to 55% in serum, and 25% in plasma (15). The proposed mechanism for this time-dependent elevation of CHI3L1 at room temperature is the degranulation of neutrophils within the blood sample. This is supported by the fact that CHI3L1 resides in the specific granules of human neutrophils and is released through exocytosis (16), consistent with the known degranulation of neutrophils and subsequent release of granular proteins during extended blood sample storage (17, 18). Concerning the effect of repetitive freeze-thaw cycles on sCHI3L1 levels, we observed that serum levels could tolerate up to three cycles without significant alteration. This aligns with previous research demonstrating CHI3L1 stability in serum, with some studies reporting no significant alteration in levels even after eight freeze-thaw cycles (15, 19). This stability is comparable to other established MS biomarkers, including neurofilament light chain (NfL) and glial fibrillary acidic protein (GFAP) (9). With this evidence at hand, and underscoring that precise preanalytical characterization is a cornerstone for biomarker clinical application, we can recommend that future practice in sCHI3L1 research to allow delayed processing of serum up to 6 h at room temperature and advise to apply a maximum of three freeze-thaw cycles.

To ensure comparability across different research centers and studies, which is a prerequisite for the clinical implementation of any biomarker, a more standardized measurement method for CHI3L1 is essential. Prior MS biomarker investigations have predominantly relied on ELISA for CHI3L1 level determinations. However, the existing variability in ELISA protocols has created challenges for direct study-to-study comparisons. This issue was recently underscored in a systematic review and meta-analysis about the role of CHI3L1 as a biomarker in MS, which noted discrepancies in the accuracy and reproducibility of various ELISA techniques for CHI3L1 quantification across different CSF and serum studies (20). To address this heterogeneity, we employed an *in-house* Simoa-based assay to determine CHI3L1 levels in serum, plasma, and CSF. This approach offered more robust quantification than commercial ELISAs and minimized experimental variability by allowing simultaneous analysis of a large number of samples for each preanalytical condition.

In conclusion, in this study we addressed critical preanalytical variables associated with sample processing and storage, to provide essential guidelines for consistent measurement. Our work reveals that serum CHI3L1 levels are highly correlated with, and therefore can be as informative as, CSF levels for tracking disease progression. Finally, the successful implementation of a novel, *in-house* Simoa-based assay provides a highly sensitive and reliable platform for quantifying CHI3L1 across different sample types, and thereby overcoming previous challenges related to assay variability. Collectively, these advancements offer valuable guidelines for sCHI3L1 assessment in clinical settings, laying crucial groundwork for its standardized and widespread integration into MS patient management.

## Data Availability

The raw data supporting the conclusions of this article will be made available by the authors, without undue reservation.
