# Supplementary material for: Impact of preanalytical factors on blood CHI3L1 levels
Source: Front Immunol. 2026 Feb 2;16:1720914. doi: 10.3389/fimmu.2025.1720914 (PMC12907172; doi:10.3389/fimmu.2025.1720914)
Supplement: Supplementary file 1 [file DataSheet1.docx]

**Supplementary material**

**Supplementary Table 1: Antibody Panel**

Antibody panel used for CHI3L1 intracellular staining of PBMCs.

| Target | Clone | Fluorochrome | Catalog | Vendor | Purpose |
| --- | --- | --- | --- | --- | --- |
| CD3 | SK7 | FITC | 344804 | Bio | Lineage |
| CD14 | MφP9 | APC | 340436 | BD |  |
| CD16 | B73.1 | PerCP-Cy™5.5 | 565421 | BD |  |
| CD19 | SJ25C1 | APC-H7 | 560177 | BD |  |
| CD45 | HI30 | BV605 | 564047 | BD |  |
| CD56 | R19-760 | BV510 | 744218 | BD |  |
| CHI3L1 |  | PE | #4815 | Quidel | Intracellular staining |
| Fixable Viability Dye |  | eF450 | 65-0863-14 | TF | Live/dead discrimination |

BD = BD Biosciences, Bio = BioLegend, TF = Thermo Fisher.

**Supplementary Table 2: Sample correlation cohort**

| Baseline characteristics | **Sample correlation cohort** |
| --- | --- |
|  |  |
| n | 30 |
| Age (years)^a^ | 39.1 (10.2) |
| Female / male (% women) | 18/12 (60.0) |
| sCHI3L1^b^ | 29.0 (17.3-36.3) |
| pCHI3L1^b^ | 13.3 (9.8-19.8) |
| cCHI3L1^b^ | 97.3 (58.4-130.1) |

^a^Data expressed as mean (standard deviation). ^b^Data are expressed as median (interquartile range). sCHI3L1: serum chitinase 3-like 1. pCHI3L1: plasma chitinase 3-like 1. cCHI3L1: cerebrospinal fluid chitinase 3-like 1. CHI3L1 levels are expressed in ng/ml

|  | **Delayed processing cohort** |
| --- | --- |
|  |  |
| n | 17 |
| Age (years)^a^ | 35.6 (8.5) |
| Female / male (% women) | 9/8 (52.9) |
| sCHI3L1 (2 h)^b^ | 20.1 (16.8-26.7) |
| sCHI3L1 (4 h)^b^ | 22.4 (16.7-30.9) |
| sCHI3L1 (6 h)^b^ | 21.9 (18.6-29.2) |
| sCHI3L1 (24 h)^b^ | 25.8 (18.6-33.3) |

**Supplementary Table 3: Delayed processing cohort**

^a^Data expressed as mean (standard deviation). ^b^Data are expressed as median (interquartile range). ^b^Median serum CHI3L1 levels in blood samples stored at room temperature for 2 h, 4 h, 6 h, and 24 h before processing. sCHI3L1: serum chitinase 3-like 1. CHI3L1 levels are expressed in ng/ml.

**Supplementary Table 4: Freeze-thaw cohort**

| Baseline characteristics | **Freeze-thaw cohort** |
| --- | --- |
|  |  |
| n | 28 |
| Age (years)^a^ | 47.7 (11.5) |
| Female / male (% women) | 12/16 (42.9) |
| sCHI3L1_Thawing cycle 1^b^ | 20.7 (15.1-28.2) |
| sCHI3L1_Thawing cycle 2^b^ | 22.3 (15.9-27.9) |
| sCHI3L1_ Thawing cycle 3^b^ | 20.2 (14.2-26.4) |
| sCHI3L1 _Thawing cycle 4^b^ | 16.6 (12.2-26.0) |

^a^Data expressed as mean (standard deviation). ^b^Data are expressed as median (interquartile range). ^b^Median serum CHI3L1 levels at different freeze-thaw cycles. sCHI3L1: serum chitinase 3-like 1. CHI3L1 levels are expressed in ng/ml.

**Supplementary Figure 1: Correlation CHI3L1 levels and age**

Supplementary Figure 1. Correlations between CHI3L1 levels across biological compartments and age. The R values represent Spearman’s rank correlation coefficient. (N=30).

**Supplementary Figure 2: Gating strategy**

Supplementary Figure 2. Gating strategies to analyze the intracellular expression of CHI3L1 in PBMCs from MS patients. (A) PBMCs were defined by SSC-Area and FSC-Area. FSC-Height vs FSC-Area were used to exclude cell aggregates from the analysis. Dead cells were excluded with a fixable viability stain. Total leukocytes were selected by gating on CD45^+^ cells. Then, CD45^+^ cells were further divided into T cells (CD3^+^), B cells (CD19^+^), monocytes (CD14^+^), NK cells (CD56^+^), and NKT cells (CD3^+^CD56^+^) subsets. Within the monocyte population, classical monocytes (cMonocytes) were defined as CD14^++^CD16^-^ and non-classical monocytes (ncMonocytes) as CD14^+^CD16^++^. The expression of CHI3L1 was then assessed in each of these cell populations
